# How Smooth Muscle Contractions Shape the Developing Enteric Nervous System

**DOI:** 10.3389/fcell.2021.678975

**Published:** 2021-06-02

**Authors:** Nicolas R. Chevalier, Richard J. Amedzrovi Agbesi, Yanis Ammouche, Sylvie Dufour

**Affiliations:** ^1^Laboratoire Matière et Systèmes Complexes, Université Paris Diderot/CNRS UMR 7057, Paris, France; ^2^Univ Paris Est Créteil, INSERM, IMRB, Créteil, France

**Keywords:** biomechanics, motility, embryo, chicken, mouse, intestine, peristalsis, neurons

## Abstract

Neurons and glia of the enteric nervous system (ENS) are constantly subject to mechanical stress stemming from contractions of the gut wall or pressure of the bolus, both in adulthood and during embryonic development. Because it is known that mechanical forces can have long reaching effects on neural growth, we investigate here how contractions of the circular smooth muscle of the gut impact morphogenesis of the developing fetal ENS, in chicken and mouse embryos. We find that the number of enteric ganglia is fixed early in development and that subsequent ENS morphogenesis consists in the anisotropic expansion of a hexagonal honeycomb (chicken) or a square (mouse) lattice, without de-novo ganglion formation. We image the deformations of the ENS during spontaneous myogenic motility and show that circular smooth muscle contractile waves induce longitudinal strain on the ENS network; we rationalize this behavior by mechanical finite element modeling of the incompressible gut wall. We find that the longitudinal anisotropy of the ENS vanishes when contractile waves are suppressed in organ culture, showing that these contractile forces play a key role in sculpting the developing ENS. We conclude by summarizing different key events in the fetal development of the ENS and the role played by mechanics in the morphogenesis of this unique nerve network.

## Introduction

The enteric nervous system (ENS) is a network of neural, glial and progenitor cells embedded inside the wall of the gastro-intestinal tract, that governs both the mechanical and chemical activity of the intestine (Costa et al., 2000). It originates from the migration of enteric neural crest cells (ENCCs) inside the developing gut during early embryogenesis. While the factors governing this early migration event have been the object of numerous investigations (Butler Tjaden and Trainor, 2013; Kang et al., 2021), we are only starting to grasp later fetal morphogenetic changes of the ENS. The ENS is comprised of two plexuses, the myenteric and submucosal plexus. Each plexus is comprised of a mesh network of glial, neural and progenitor neural crest cells condensed in ganglia, that are interconnected by inter-ganglionic, axonal nerve fibers. The myenteric plexus is sandwiched between the longitudinal and the circular smooth layer, and projects neurites to smooth muscle and is interconnected with the submucosal plexus (Chevalier et al., 2019); the submucosal plexus is located right above the mucosa and projects to the epithelium. Unlike the central nervous system which is protected by a skull or a spine, the ENS is embedded in a soft, deformable tissue and is constantly subject to mechanical stress, both during development and in homeostasis in the adult. Mechanical stress is one of the essential physiological inputs mechanosensitive neurons in the gut respond to Mazzuoli-Weber and Schemann (2015). Mechanical constraints have a strong influence on the growth of neurons in culture (Franze, 2013): applying a constant mechanical stretch to axons has for example been shown to induce axonal growth of up to 8 mm per day (Pfister, 2004); axons in neural networks spontaneously generate mechanical tension and this tension in return actively stabilizes the axon (Anava et al., 2009); minute externally applied mechanical forces modify axon growth direction (Bray, 1979). In-vivo, mechanical forces have recently been shown to mediate retrograde axon extension in the developing zebrafish olfactory bulb (Breau et al., 2017). The main source of mechanical stress acting on the ENS during fetal development is due to spontaneous contractions of the circular smooth muscle (Holmberg et al., 2004; Roberts et al., 2010; Chevalier et al., 2017). These contractions start as early as embryonic day 6 (E6) in the chicken embryo (Chevalier et al., 2017), and E13.5 in the mouse (Roberts et al., 2010), they take on the form of waves that propagate longitudinally along the gut at a frequency of 1–5 cycles per minute and with increasing amplitude as the intestine matures. This early muscular activity is independent of neural input (Roberts et al., 2010; Chevalier et al., 2019). The goal of this report is to investigate the effects of this smooth-muscle generated mechanical stress on the morphogenesis of the ENS in chicken and mice. We present data on the morphological evolution of the ENS in those species at fetal stages, examine the deformation pattern generated on the ENS by the circular contractile waves, and the effect of suppressing the waves in organ culture on ENS morphogenesis.

## Results

### Morphogenesis of the Chicken Enteric Nervous System Occurs by Anisotropic Expansion of a Hexagonal Honeycomb Mesh Without *de-novo* Ganglion Formation

**FIGURE 1:**
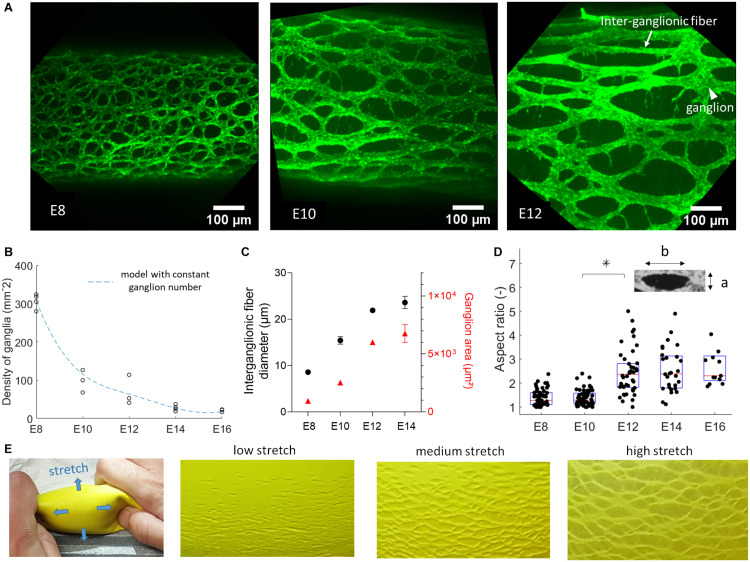
Fetal development of the chicken enteric nervous system. **(A)** Wholemount immunolabeling with Tuj1 antibody against βIII-tubulin showing the evolution from E8 to E12 of the neuronal network of the ENS in the duodenum, same scale images. The rostro-caudal axis is horizontal in all images. **(B)** Experimental evolution of the number of ganglia per mm^2^ in the duodenum, one dot per sample, sample numbers are E8 *n* = 4, E10 *n* = 3, E12 *n* = 3, E14 *n* = 4, E16 *n* = 3. Dashed blue line: model prediction assuming total number of ganglia is set at E8. **(C)** Inter-ganglionic fiber diameter (black, left axis) and average ganglion area (red, right axis), *n* = 3 different guts for each age, error bar length is SD, when no error bar is visible it is smaller than dot size. For each sample, the dimensions of *n* = 6 fibers and ganglia were measured and averaged. **(D)** Aspect ratio b/a (inset) of inter-ganglionic spaces, one dot per inter-ganglionic space analyzed. **(E)** A gel surrounded by a rubber membrane is pressurized, causing stretch along the blue arrows. At low stretch, defects (small cracks) in the rubber are small and dense. As the membrane stretches out, the cracks move apart but their total number does not change, providing a visual analogy to chicken fetal ENS development.

As from embryonic day 8, the chicken GI tract is entirely colonized by ENCCs; in the myenteric plexus (Figure 1A), these cells have condensed to ganglia (mesh nodes) connected to each other by inter-ganglionic fibers (internodes). Each ganglion consists of several neural, glial and progenitor cells, and is typically connected to 3 or 4 ganglia in its immediate vicinity. The inter-ganglionic fibers consist of axons and glial cells. A suitable geometric model of this mesh is a honeycomb structure (Mihai and Goriely, 2014) with either a hexagonal structure (each ganglion has connections to 3 neighboring ganglia) or a diamond structure (4 neighboring ganglia). As development progresses, the size of ganglia, fibers, and of the inter-ganglionic spaces increases (Figure 1A). The area density of ganglia (number of nodes per surface area) decreases from 300 ganglia/mm^2^ at E8 to 20 ganglia/mm^2^ at E16 (Figure 1B). The average size of ganglia and of inter-ganglionic fibers increases with age (Figure 1C). We further found that the ENS mesh becomes more elongated along the longitudinal direction, as shown by measuring the aspect ratio of inter-ganglionic spaces from E8 to E16 (Figure 1D).

The decrease of ganglion density can be explained by considering that the total number of ganglia is set at E8, and that they subsequently become more spaced out as the total surface area of the intestine increases because of tissue growth. We tested this hypothesis by computing σ(*t*) = σ_*E*8_*A*_*E*8_/*A*(*t*), where σ_*E*8_ = 300 mm^–2^ is the experimental ganglion density at stage E8, σ(*t*) the predicted ganglion density at a later stage *t*, *A*_*E8*_ and *A*(*t*) the total surface area of the intestine at stage E8 and a later stage *t* respectively. The surface area of the duodenum in the period E8-E16 was obtained from diameter and length data compiled by Coulombres and Coulombres (1958) on *n* = 10 chicken embryos per age group. The predicted ganglion density σ(*t*)is represented by the dashed blue curve in Figure 1B, and provides an excellent fit to the experimentally measured densities. This provides evidence for the fact that the total ganglion number of the chicken gut is set shortly after gangliogenesis at E8 and that, although individual ganglia and fibers continue to grow, there is no *de novo* ganglion formation after this stage. At E8, the gut is 20 ± 3 mm long (from duodenum to hindgut, *n* = 23) and has a diameter of 0.38 ± 0.06 mm (*n* = 23); from the ganglion density of 300 mm^–2^ at this stage (Figure 1B), we find a total ganglion number of 5000 to 9500 for the lower gastrointestinal tract of the chicken. Because this number is high, we could not resort to individual counting of all ganglia to confirm that it remains constant throughout development, but believe that the model fit provided in Figure 1B provides strong evidence thereof. Subsequent anisotropic growth (lengthening) of the intestinal tissue increases the distance between ganglia (Figures 1A,B), and orients the ENS in the longitudinal direction (Figures 1A,D). The increase in anisotropy of the ENS becomes most pronounced between stages E10 and E14 (Figure 1D), precisely when longitudinal growth of the intestine is most vigorous (Khalipina et al., 2019). A physical toy model of this morphogenesis route is shown in Figure 1E. It consists of a rubber sheet presenting small cracks. When the sheet is stretched, the distance between cracks increases; their surface density therefore decreases, providing a visual analogy to the morphogenesis of the chicken ENS (Figure 1A).

### Circular Smooth Muscle Contractions Induce a Longitudinal Strain on the ENS

**FIGURE 2:**
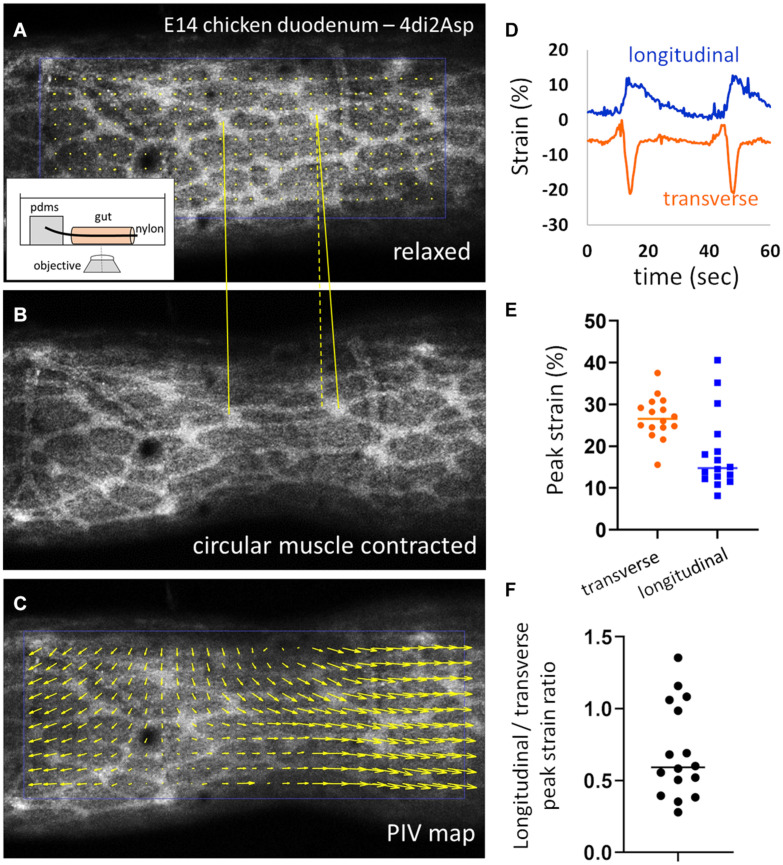
Circular smooth muscle contractions induce a longitudinal strain component. **(A,B)** Frames from Supplementary Video 1 in the relaxed state and during a CSM contraction. Inset: experimental setup. **(C)** PIV map of displacement vectors retrieved from images **(A)** and **(B)**. **(D)** Representative longitudinal and transverse strain measured by ganglion tracking during two contraction events, the transverse strain is negative (compression) and the longitudinal strain positive (elongation). **(E)** Absolute values of peak transverse and longitudinal strain and **(F)** absolute peak longitudinal over peak transverse strain ratio measured for 16 contractions from *n* = 6 E14 duodenums.

We next investigated the cause of the anisotropic, longitudinal growth of the ENS. We previously showed (Khalipina et al., 2019) that during gut embryogenesis, spontaneous contractions of the circular smooth muscle layer induce lengthening of the whole intestine, because the circumferential “squeezing” induces strain along the longitudinal direction. This is called a Poisson effect in material science. We were interested in revealing specifically the effect of the contractions on the deformation of the ENS. To image the chicken ENS while keeping the organ alive we used the fluorescent vital dye 4-Di-2-Asp (Hanani, 1992). This dye stains nerve fibers, and possibly also glial cells; it has been suggested that it targets the mitochondrial and plasma membrane of excitable cells but the precise mechanism of staining is not known (Hanani, 1992; Cornelissen et al., 1996). We noticed that staining with 4-Di-2-Asp could reveal the ENS only as from E12, and that the quality (sharpness) of staining increased with age. Neurons are already present and differentiated throughout the gut as from E8 but staining with 4-Di-2-Asp yielded a uniform, non-specific staining of the gut tissue for guts younger than E12. This difference of staining at different stages may reflect the level of electrical and mitochondrial activity of the neurons; we have indeed previously shown that the first signs of neuromuscular activity in the embryonic chicken gut only appear at E14-E16 (Chevalier et al., 2019). Stained guts were mounted on thin glass rods inserted in the lumen and we made sure the bottom of the gut did not contact the dish (see section “Materials and Methods” and insert Figure 2A). This friction otherwise perturbs the physiological deformation of the organ when imaging from below. We imaged them with a confocal microscope, in physiological conditions (37°C, carbogen-saturated DMEM). Supplementary Video 1 shows a typical example of ENS deformations induced by spontaneous contractions as seen using the 4-Di-2-asp stain. Figures 2A,B show the ENS in E14 chicken duodenum respectively before the onset of a circular smooth muscle contraction, and during the contraction. In addition to the transverse component, which makes the inter-ganglionic fibers come closer together, the contraction also pulls ganglia apart in the longitudinal direction (yellow lines). We computed the displacements and strains acting on ENS structures.

The transverse and longitudinal displacements induced by the contraction is clearly revealed when performing particle-velocimetry analysis (Figure 2C, Materials and Methods). In order to compute the strains, we tracked the change in length of a segment that connected two ganglia either transversely or longitudinally, using the Tracker ImageJ plugin (Materials and Methods); we do not have access to radial strain in this experimental setup. Typical strain components recovered by this method are illustrated in Figure 2D: the transverse strain is negative (corresponding to compression along this direction) and the longitudinal strain positive (elongation along this direction). We measured absolute peak transverse strains of 28 ± 5 % (all values expressed as ± SD) and peak longitudinal strains of 18 ± 9 %, for *n* = 16 contractions analyzed from *n* = 6 different E14.5 duodenum samples (different guts, Figure 2E). The absolute ratio of the longitudinal strain over the transverse strain varied widely from 0.27 to 1.35, with a mean 0.70 ± 0.33 (Figure 2F). Supplementary Video 2 shows an example of a contraction triggering very strong longitudinal strain on ENS inter-ganglionic fibers. We confirmed that the same qualitative circumferential compression & longitudinal stretch happens at earlier stages as well (E12, Supplementary Video 3), when the anisotropy of the ENS becomes most pronounced (Figure 1D).

### Mechanical Finite Element Simulation Predicts Longitudinal Strain Across All Gut Layers

**FIGURE 3:**
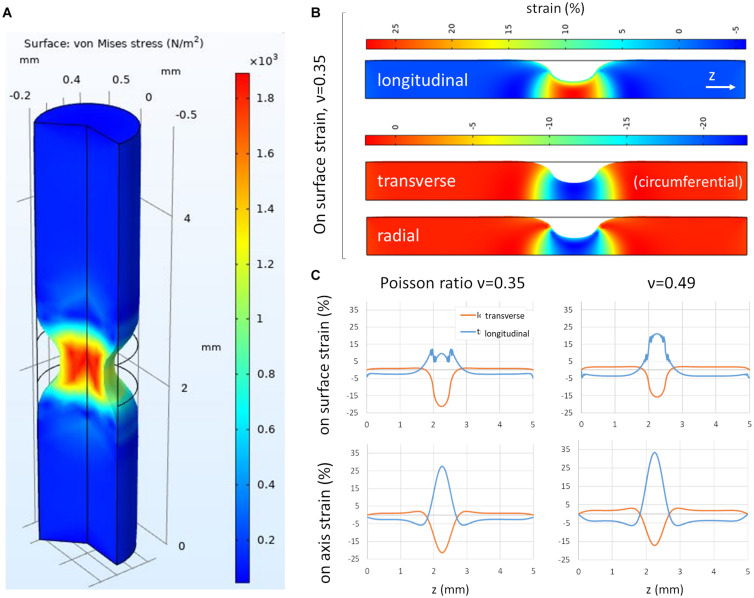
Finite element modeling of the gut subject to a circular contraction. **(A)** 3D view of von Mises stress, **(B)** longitudinal, transverse and radial stress, 2D view (of 3D axisymmetric model), **(C)** Transverse and longitudinal strain on gut surface and on gut axis for two values of the Poisson ratio ν = 0.35 and ν = 0.49, as a function of longitudinal position along the gut cylinder.

To gain a better understanding of the different strain components induced by CSM contractions, we set up a finite element model of the gut, assimilated to a full cylinder (the lumen only represents ∼10% of the transverse section of the gut at E14), locally compressed by an inward directed (radial), centripetal force (Figure 3A). The longitudinal, transverse and radial strain components induced by this compression are illustrated in Figure 3B. While the transverse compressive strain corresponds to a reduction of the gut circumference, the radial compressive strain induces thinning of the gut wall during a contraction; we cannot read out the latter from our experimental setup (Figure 2). We experimentally determined the Poisson ratio of the gut at E10 to be ν = 0.36 ± 0.09 (*n* = 7) (Khalipina et al., 2019). This ratio was measured by applying fixed longitudinal strain *s_l_* on the whole gut, measuring the resulting reduction in diameter *s_d_* and computed as ν = *s*_*d*_/*s*_*l*_. We found that for a Poisson ratio of ν = 0.35, the longitudinal over transverse strain ratio at the surface of the gut (where the ENS is located) was 0.5, and it increased to 1.25 for ν = 0.49 (Figure 3C). These values are consistent with the ones we find experimentally (0.27–1.35, Figure 2F). The simulation further allowed us to get insight into the magnitude of the Poisson effect inside the gut. We found that on the cylinder axis, the Poisson effect was even higher, with longitudinal over transverse strain ratio of 1.35 (ν = 0.35) and 1.94 (ν = 0.49) (Figure 3C bottom panels). We therefore expect that not only the periphery of the gut be subject to this longitudinal strain, but also the more interior submucosal and epithelial layers.

We further considered how the presence of a lumen affects the strain patterns. We considered a lumen area 20% of the total section area of the gut, which is representative of the size of the lumen at age E14 (Savin et al., 2011). We found that surface transverse strains increased (+25%) at the surface compared to the no-lumen case, whereas surface longitudinal strains did not change (Supplementary Figure 1). Resulting absolute longitudinal over transverse strain ratio at the surface of the gut were therefore 20% lower (0.4 for ν = 0.35) than in the no-lumen case (0.5 for ν = 0.35). In the no-lumen case, the longitudinal strain increases as one penetrates radially deeper inside the tissue (toward the epithelium), while the transverse strain remains constant (Figure 3B). When considering a lumen (Supplementary Figure 1), both the longitudinal stretch and transverse compression increase as one travels radially toward the epithelium; the resulting absolute longitudinal over transverse strain ratio at the level of the epithelium was 0.8 (for ν = 0.35), which is less than in the case with a lumen (1.35 for ν = 0.35), but still higher than at the surface of the intestine. All in all, considering a small lumen does not qualitatively change the conclusions reached from the no-lumen case Figure 3.

### Inhibiting Circular Smooth Muscle Contractions in Culture Yields Isotropic ENS Growth

**FIGURE 4:**
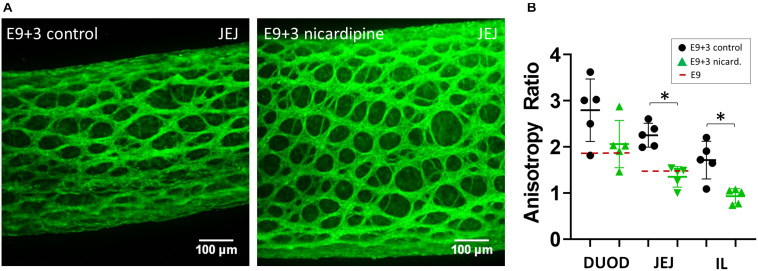
Nicardipine inhibits longitudinal anisotropic growth of the ENS. **(A)** Representative Tuj1 immunostaining (max z-projection of stack) for control (vehicle only) samples and nicardipine treated samples after 3 days in culture (E9+3). DUOD, duodenum; JEJ, jejunum; IL, ileum. **(B)** ENS aspect ratio of control and nicardipine treated samples. Each dot is the average of the aspect ratio of 5–7 inter-ganglionic spaces from a different gut (different embryo). The dashed red line indicates the aspect ratio at E9. ^∗^: statistically significant difference, Mann-Whitney two-tailed test, *p* < 0.05.

Because circular contractions were seen to stretch the inter-ganglionic fibers, we posited that they could, incrementally, over developmental times, cause the anisotropic growth of the ENS reported in Figure 1. To test this hypothesis, we evaluated the effect of suppressing contractions with the L-type Ca^2+^ channel blocker nicardipine on the evolution of ENS morphology in culture. We had previously shown that nicardipine strongly reduces contractions in chicken embryonic gut (Khalipina et al., 2019). We measured the average aspect ratio from 5 to 7 inter-ganglionic spaces from *n* = 5 different guts (embryos) for each condition (vehicle or nicardipine). Nicardipine-treated samples exhibited a much reduced anisotropy of ganglionic structures compared to controls after 3 days in culture (E9+3, Figure 4A). The ENS aspect ratio of E9+3 samples treated with nicardipine was equal to that before culture, at E9 (Figures 1D, 4B) in the three regions analyzed, whereas the anisotropy in control samples had significantly increased compared to their pre-culture levels. These results are in agreement with images presented by Huycke et al. (2019). We can therefore conclude that the contractions play a determining role in driving anisotropic growth of the entire gut (Khalipina et al., 2019), and also of cellular networks like the ENS within the gut.

### Identification of Longitudinal Strain and Developmental Effects in the Mouse ENS

**FIGURE 5:**
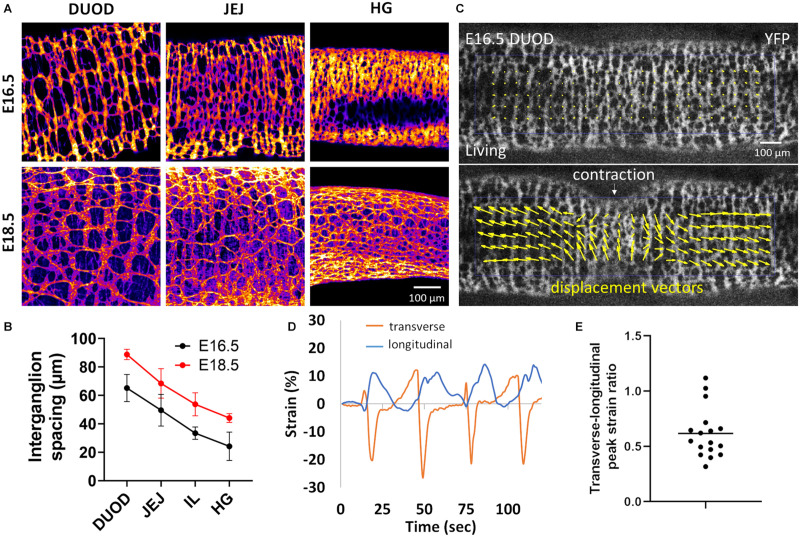
Longitudinal stretch by circular contractions induces longitudinal spacing of enteric ganglia in the mouse. **(A)** Tuj1 immunostaining of the mouse ENS at E16.5 and E18.5 in the duodenum, jejunum and hindgut (HG). Ganglia are clearly seen to be more spaced apart in the longitudinal (horizontal) direction with increasing age. The scale bar of all panels is the same as in the bottom right corner. **(B)** Average inter-ganglion spacing, each dot is the average of the spacing measured for *n* = 4 embryos at each stage, error bar is ± SD. **(C)** Frame from Supplementary Video 4 in the relaxed state and during a CSM contraction, E16.5 mouse duodenum, lower panel: PIV map of displacement vectors. **(D)** Representative longitudinal and transverse strain measured by ganglion tracking of four successive contraction events. **(E)** Peak longitudinal over peak transverse strain ratio measured for 17 contractions from *n* = 4 E16.5 duodenums.

We wondered whether a similar “longitudinal stretch induced by circular contractions” mechanism might govern the morphogenesis of the ENS in the mouse. Unlike the chicken ENS, the fetal mouse ENS is organized in a rectangular grid structure (Newgreen et al., 2013), with ganglia stretched circumferentially and inter-ganglionic fibers running longitudinally. We have shown (Chevalier et al., 2020) that this structure results from the adhesion and reorientation of ENCCs to circumferentially spun extra-cellular matrix fibers produced by the circular smooth muscle. This orientation of ganglia takes places in the period E14.5-E16.5 in the mouse midgut. Because the mouse ENS presents a square lattice we measured the longitudinal average inter-ganglion spacing (from Tuj1 immunostained wholemount guts) as a measure of anisotropy; measuring the aspect ratio of interganglionic spaces is also possible and would provide similar results (Figure 5A). Figures 5A,B show that the longitudinal average inter-ganglion spacing increases by 36% in the duodenum, 38% in the jejunum, 61% in the ileum and 82% in the hindgut. To examine how spontaneous circular contractions deform the murine ENS, we worked with a genetically modified mouse strain that expresses a fluorescent YFP reporter in all ENCCs (see Materials and Methods); we then applied the same methodology as for chicken (Figure 2) to estimate the strains on ENS structures. PIV mapping (Figure 5C and Supplementary Video 4) shows that both transverse and longitudinal displacements take place during circular contractions, which we could quantify with the Tracker plugin (Figure 5D). We measured peak transverse strains of 23 ± 3 % and peak longitudinal strains of 14 ± 6 % for *n* = 17 contractions analyzed from *n* = 4 different duodenum samples (different guts). The ratio of the longitudinal strain over the transverse strain (Figure 5E) was in the range 0.4 to 1.12, with a mean 0.62 ± 0.26. These results show that circular contractions also induce longitudinal strain on the mouse ENS and on the murine gut as a whole.

## Discussion

**FIGURE 6:**
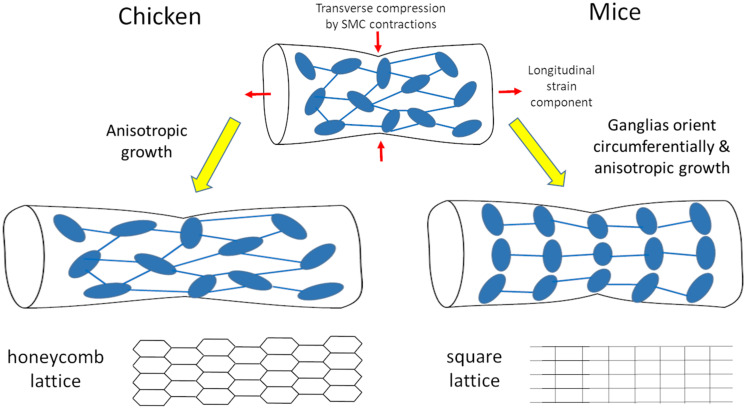
Scheme of morphogenesis routes for the chicken and mouse enteric nervous system resulting respectively in honeycomb and square lattices.

We have shown that morphogenesis of the fetal chicken ENS consists in the anisotropic longitudinal expansion of a honeycomb network. The initial ganglion density of this network is high at E8, and decreases as the gut expands, without de-novo ganglion formation (Figure 1). Spontaneous circular contractions start as soon as circular smooth muscle differentiates at E5-E6 in the chicken (Chevalier et al., 2017), E14.5 in the mouse (Roberts et al., 2010). The contractions increase in amplitude and frequency as the smooth muscle layer develops (Chevalier et al., 2017). The longitudinal anisotropy of the ENS is a consequence of the longitudinal stretch imposed by spontaneous contractions of the circular smooth muscle (Figures 2, 3); abrogating this mechanical component results in isotropic ENS expansion (Figure 4). We have found that mechanical stresses induced by contractions in the developing mouse ENS are similar to those in the chicken ENS, resulting in anisotropic expansion of the murine gut, and increased spacing of enteric ganglia in the longitudinal direction as development proceeds (Figure 5). Figure 6 summarizes these important morphogenetic transformations taking place in the fetal gut of mice and chicken.

The hexagonal honeycomb geometry of the chicken myenteric plexus is set as ganglia are formed at E7-E8 (Figure 1). The spontaneous emergence of this spatial arrangement might result from repulsive biochemical interactions between ganglia, a hexagonal lattice geometry being the minimal energy configuration toward which repelling particles self-assemble (Merminod et al., 2014). Because the intestine grows and the number of ganglia remains constant, their density decreases, they become more spaced out. We note that there are quantitative, but not qualitative differences in the ENS growth mechanism we describe between duodenum, jejunum or ileum. Development of the muscle and of the ENS proceeds rostro-caudally in the midgut: at a given age, fiber diameter, ganglion size and ganglion aspect ratio are higher in the duodenum than in the jejunum, while the ganglion density is lower (Figures 4, 5). Similar data on the decrease of ganglion density with age has been reported in rats (Schäfer et al., 1999; Vries et al., 2010). The decreased density is also clearly seen on whole-mount labeling of postnatal mouse (Breau et al., 2009; Hatch and Mukouyama, 2015) and human (Wester et al., 1999) myenteric plexus. As from ∼E10, thin neurites extending circumferentially from the ganglia grow to innervate the circular smooth muscle layer (Chevalier et al., 2019).

We have shown that abrogating contractility in the chicken embryonic gut abolishes the anisotropic growth of the gut (Khalipina et al., 2019) and of the ENS (Figures 1, 4). We have not performed the same experiment in mice because we did not have the proper protocol to grow embryonic mice guts in culture. Yang et al. (2020) have however recently performed refined genetic manipulations that demonstrates how disrupting the organization of smooth muscle cells by three independent means systematically reduces gut elongation in-vivo. Together, these results strongly suggest that the same “longitudinal stretch induced by circular contractions” mechanism that yields anisotropic growth of the gut and of the ENS in the chicken is at play during fetal morphogenesis of the murine gut and of its ENS. It has recently been shown (Huycke et al., 2019) that local, static circumferential residual stress determines the orientation of circular smooth muscle in the developing chicken gut. We suggest that the longitudinal stretch component associated to the circular contractions could also explain why the outer smooth muscle layer (located right above the ENS) that differentiates at E13 in the chicken and E16.5 in the mouse is longitudinally oriented. This view provides an alternative, simpler scenario than the explanation laid out by Huycke and colleagues (Huycke et al., 2019), which relies on a difference between the mechano-biological orientation effects of static and cyclic stretch.

Enteric neurons are intrinsically mechanosensitive (Mazzuoli and Schemann, 2012), and it is therefore important to understand the type of stress they are subject to, both during embryonic development, and in adult gut motility. Mechanical force has been put forward as a chronic source of stimulation, nay stress of the ENS. This could be a reason for the important turnover rate of the ENS in the adult (Kulkarni et al., 2017). It should be noted that there is some controversy around this, other investigators having found that there is little or no neurogenesis in the adult mouse gut (Joseph et al., 2011). Data on in- or ex-vivo deformations of the ENS mesh are scarce; the study by Gabella and Trigg (1984) is the only one to present deformations of a ganglion in different contractile states, after tissue fixation. To the best of our knowledge, Supplementary Videos 1–5 and Figures 2, 5 therefore represent the first live recordings of the mechanical strain the ENS is subject to during physiologic spontaneous smooth muscle activity. Working on embryonic guts presents the distinctive advantage that the gut is small and thus the deformation of the whole ENS during motility events can be monitored; the embryonic gut can be used as a “miniature model” to infer the deformations in the adult gut. Because the geometry of the ENS differs between the chicken and the mouse, the ganglia and inter-ganglionic fibers are subject to slightly different strains during circular smooth muscle contractions in these two species. In the chicken, the angles formed by two inter-ganglionic fibers connected to the same ganglion became sharper at the site of the contraction (Supplementary Videos 1, 2); this change of angles absorbs some of the deformation, like upon deformation of a hexagonal lattice (scheme Figure 6). In the mouse, circular contractions caused a pronounced compression of the circularly oriented ganglia (Supplementary Videos 4, 5, along with a longitudinal stretch of inter-ganglionic fibers, as reported in Figure 5. Circular contractions are however, only one type of stress the gut is subject to. We expect for example that the presence of bolus in the adult gut will stretch out the ENS in both lattice types. It is unclear at this stage whether these difference in geometries have a functional implication. In humans, a rectangular myenteric plexus has been revealed by NADPH-diaphorase staining in the small bowel (midgut) of full-term newborns (Nemeth et al., 2000). However, other images of the myenteric plexus of the small bowel in a 10-day old infant, a 9-year old child (Wester et al., 1999), and in a specimen reported by Auerbach himself (Furness, 2007) show a geometry that seems closer to a hexagonal mesh. There might therefore be some regional differences in the geometry of the myenteric neural mesh in the human midgut. The myenteric plexus of the human colon has a hexagonal honeycomb geometry (Wedel et al., 1999; Wester et al., 1999; Hanani et al., 2012); human and chicken myenteric plexuses are in fact morphologically strikingly similar in the hindgut.

The example of the ENS shows distinctly how mechanical growth responses of neurons evidenced ex-vivo (Bray, 1979; Pfister, 2004) are also at play during organogenesis. Future research efforts will be aimed at clarifying the roles of mechanical stress on cellular homeostasis of the adult ENS (Kulkarni et al., 2017) and in the genesis of intestinal motor patterns (Spencer et al., 2016; Chevalier et al., 2019).

## Materials and Methods

### Ethics

All procedures presented in this manuscript have been approved by the ethics committee ComEth Anses/ENVA/UPEC, APAFiS request number 2019061218208141 (#210725). All experiments were performed in accordance with the ethics guidelines of the INSERM and CNRS.

### Specimens

Fertilized chicken eggs were purchased from EARL Morizeau (Chartres, France, breeder Hubbard, JA57 hen, I66 rooster, yielding type 657 chicks). The eggs were incubated at 37.5°C in a humidified chamber for 8–16 days. The gastrointestinal tract was dissected out from the embryos from hindgut to proventrilicus and the mesentery carefully removed.

Mouse models used in this study are the Cre reporter mice Gt(ROSA)26Sortm1(EYFP)Cos (Srinivas et al., 2001), for EYFP, and B6.Cg-Gt(ROSA)26Sortm9(CAG-tdTomato)Hze/J (Madisen et al., 2010) for tdTomato are refereed as YFPfl and Tomatofl/fl, respectively. Finally, Tg(PLAT-cre)116Sdu (Pietri et al., 2003) a transgenic mouse line in which the transgene is under the control of the 3-kb fragment of the human tissue plasminogen activator (Ht-PA) promoter is referred as Ht-PA::Cre. Ht-PA::Cre were crossed with mice heterozygous for the Itgb1 (beta1neo/+) to generate Ht-PA::Cre; beta1neo/+. YFPfl/fl orCre reporter mice Tomatofl/fl were cross with Ht-PA::Cre mice to generate embryos where YFP or tdTomato are specifically expressed into the NCC and their derivatives carrying beta1fl/fl to generate YFPfl/fl; beta1fl/fl. Embryos carrying the expression of fluorescent reporter proteins in the context of wild type or the conditional mutation for Itgb1 in NCC were generated as previously described (Breau et al., 2009). The day of the vaginal plug was considered E0.5. The gastrointestinal tract of mice embryos was dissected from hindgut to stomach and the mesentery carefully removed.

### Monitoring of ENS Deformations

For monitoring ENS deformations under the effect of spontaneous smooth muscle contractions, E14-E16 chicken guts were stained for 30 min in 4 μM 4-Di-2-Asp in PBS at room temperature (RT) and washed for 2 h in PBS at RT. Mice guts with fluorescently-tagged ENCCs were dissected, the duodenum was isolated and were used as such. Gut segments (∼1 cm) were mounted on thin glass rods that we inserted in the lumen. The rods were pinned horizontally, at a distance of ∼0.5 mm from the dish bottom, so that the gut did not contact the dish bottom. In this way the bottom of the gut is free to move and the deformations induced by spontaneous contractions could be imaged using an inverted confocal microscope. Motility was recorded at magnification ×4 or ×10, using GFP filter sets on an Olympus spinning disk confocal microscope, at a 1 Hz time-lapse frequency, in 5 mL of carbogen-saturated DMEM at 37°C. No z-stack imaging was performed during the time-lapse.

### Organotypic Culture

E9 chicken guts were cultured in 1 mL of DMEM GlutaMAX^TM^ -I (Thermofisher, with 4.5 g/L D-glucose and sodium pyruvate, Ca^2+^ 1.8 mM, Mg^2+^ 0.8 mM) with 1% penicillin-streptomycin in a 35mm diameter Petri dish for 72 h in a humidified incubator at 37°C, in a 5% CO_2_ – 95% O_2_ (carbogen) atmosphere, with one medium change after 36 h. We had previously shown that this method allowed growth of the guts (Khalipina et al., 2019). After 72 h, the guts were processed for whole-mount immunohistochemistry.

### Immunohistochemistry

Guts (E8-E16) were fixed for 1 h in a 4% PFA in PBS solution, then blocked and permeated in 1% BSA and 0.1% triton in PBS overnight, immersed in anti βIII-tubulin antibody (Abcam, ref14545, dilution 1:1000) for one day, washed and immersed in Alexa488 secondary antibody (dilution 1:400) for at least 4 h, and imaged using a confocal CSU-W1 Zeiss spinning disk confocal microscope.

### Image Analysis

Z-stacks of the chicken ENS were max-projected along the z-axis, thresholded and the average pixel value retrieved with ImageJ the density of Tuj1. Ganglia were counted manually and the ganglion density obtained by dividing by the area (half cylindrical surface) of the gut.

For PIV (particle image velocimetry), we used the open-access PIVlab Matlab code, courtesy of Thielicke and Stamhuis (2014). The displacement of a given point $\vec{x_{1}}$ after a time Δ*t* is $\Delta \vec{x_{1}}=\vec{x_{1}}\left(t+\Delta t\right)-\vec{x_{1}}\left(t\right)$. We define the strain $\vec{s}$ acting on a segment $\vec{x_{2}}-$$\vec{x_{1}}$ (for example an interganglionic fiber located between two ganglia at $\vec{x_{2}}$ and $\vec{x_{1}}$) is the relative change in length of this segment between the relaxed state at time *t* and the contracted state at time *t* + Δ*t*,

$$\begin{array}{c} \vec{s}=\frac{\left(\vec{x_{2}}\left(t+\Delta t\right)-\vec{x_{1}}\left(t+\Delta t\right)\right)-\left(\vec{x_{2}}\left(t\right)-\vec{x_{1}}\left(t\right)\right)}{\left(\vec{x_{2}}\left(t\right)-\vec{x_{1}}\left(t\right)\right)}\\ \;=\frac{\Delta \vec{x_{2}}-\Delta \vec{x_{1}}}{\left(\vec{x_{2}}\left(t\right)-\vec{x_{1}}\left(t\right)\right)}. \end{array}$$

To retrieve the transverse and longitudinal strains, particle tracking was performed with the Tracker ImageJ plugin developed by O. Cardoso; we chose two ENS nodes (ganglia) located either longitudinally of transversely to track and compute the deformations along these two directions.

### Finite Element Simulation

The study was implemented in COMSOL Multiphysics 5.4 and was modeled in the 2D axisymmetric steady solid mechanics module. A full cylinder (5 mm long with a radius 0.5 mm) is fixed at extremities and a localized force density is applied to study the strain and stress that evolve at different Poisson ratios (0.1–0.49). The material used is isotropic and has a Young’s Modulus of 5 kPa. For modeling the contraction, a force density is applied to the wall of the cylinder, in the radial direction, with a Gaussian distribution of width 0.2 mm localized at the middle of the gut. The amplitude of the force was chosen to reflect physiological transverse deformations. The finest mesh size available from the modeling toolbox was used. Boundary conditions: the coordinate-dependent load described serves as the boundary condition at the tube’s outer surface. The top and bottom ends of the tube are fixed along both z and r coordinate axes.

## Data Availability Statement

The original contributions presented in the study are included in the article/Supplementary Material, further inquiries can be directed to the corresponding author/s.

## Ethics Statement

The animal study was reviewed and approved by ComEth Anses/ENVA/UPEC.

## Author Contributions

NC performed experiments, conceptualized and supervised the research, produced figures, and wrote the manuscript. RA performed finite element simulations and produced figures. YA performed experiments, and quantitative analysis and produced figures. SD genetically engineered the mouse strains, and provided samples and critically revised the manuscript. All authors contributed to the article and approved the submitted version.

## Conflict of Interest

The authors declare that the research was conducted in the absence of any commercial or financial relationships that could be construed as a potential conflict of interest.

## Supplementary Material

Supplementary Video 1 Time-lapse of 4-Di-2-Asp fluorescently labeled E14 myenteric plexus of chicken duodenum, during spontaneous contractions of the circular smooth muscle, analyzed in Figure 2.

Supplementary Video 2 Time-lapse of deformations of 4-Di-2-Asp fluorescently labeled E16 myenteric plexus of chicken duodenum, during spontaneous contractions of the circular smooth muscle, showing extreme longitudinal strain exerted on inter-ganglionic fibers and the change of the angle between inter-ganglionic fibers stemming from one ganglia.

Supplementary Video 3 Time-lapse of deformations of 4-Di-2-Asp fluorescently labeled E12 myenteric plexus of chicken duodenum, with displacement vectors from PIV analysis. Longitudinal stretch of the ENS is also clearly seen at this embryonic stage.

Supplementary Video 4 Time-lapse of the YFP+ myenteric plexus of E16.5 mouse jejunum during spontaneous contractions of the circular smooth muscle, magnification ×4.

Supplementary Video 5 Time-lapse of the YFP+ myenteric plexus of E16.5 mouse jejunum during spontaneous contractions of the circular smooth muscle, magnification ×10, showing prominent transverse strain on circularly oriented ganglia. Intensity changes are due to motion of the ENS in and out of the confocal imaging plane.
