# Supplementary material for: How Smooth Muscle Contractions Shape the Developing Enteric Nervous System
Source: Front Cell Dev Biol. 2021 Jun 2;9:678975. doi: 10.3389/fcell.2021.678975 (PMC8206791; doi:10.3389/fcell.2021.678975)
Supplement: Supplementary file 6 [file Data_Sheet_1.PDF]

Supplementary Information to:

How smooth muscle contractions shape the developing enteric nervous system

Nicolas R. Chevalier[1*], Richard J. Amedzrovi Agbesi[1†], Yanis Ammouche[1†], Sylvie Dufour[2]

[1] *Laboratoire Matière et Systèmes Complexes, Université Paris Diderot/CNRS UMR 7057, Sorbonne Paris Cité, 10 rue Alice Domon et Léonie Duquet, 75013 Paris, France*

[2] *Univ Paris Est Créteil, INSERM, IMRB, F-94010 Créteil, France*

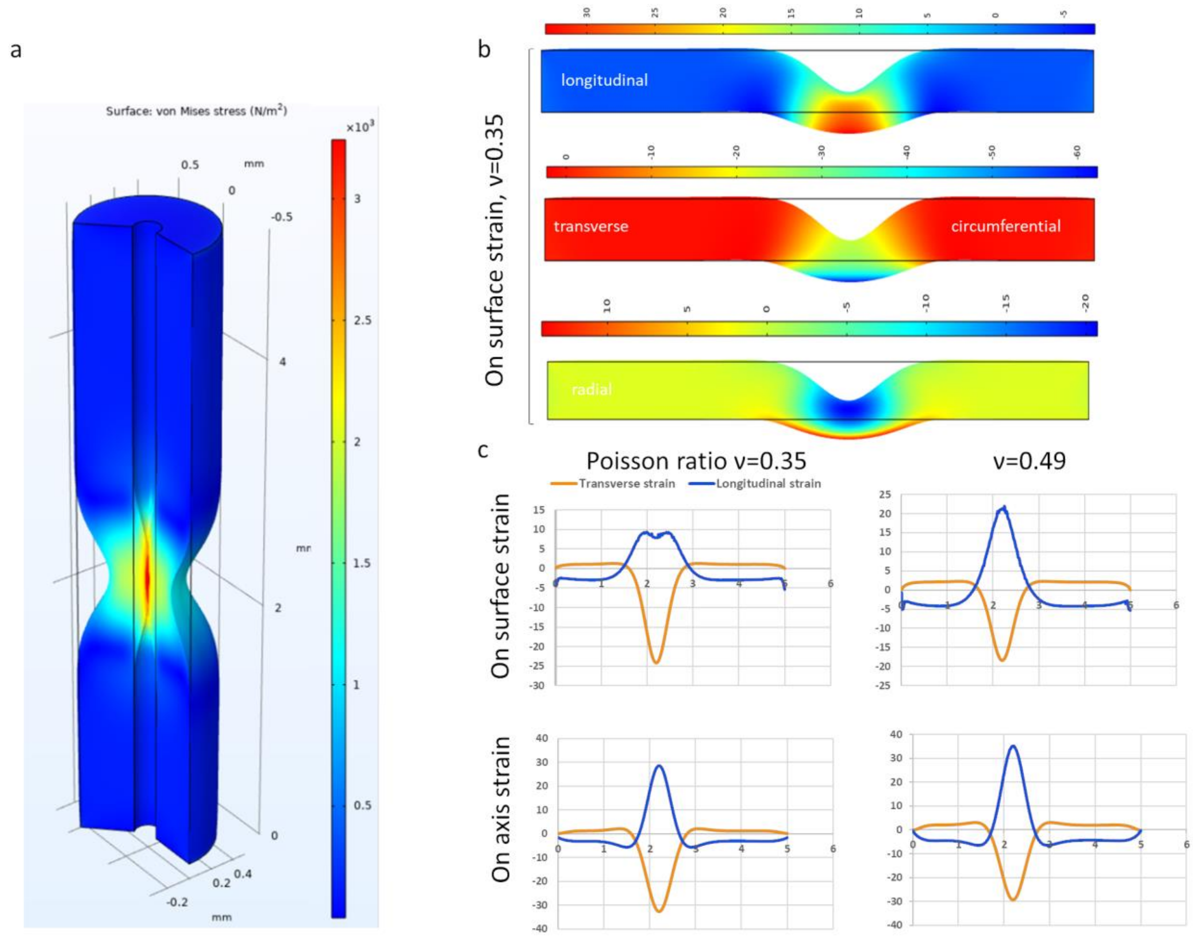

Figure S1. Finite element modeling of the gut subject to a circular contraction, including a lumen 20% of the cross-section of the full gut. (a) 3D view of von Mises stress, (b) longitudinal, transverse and radial stress, 2D view (of 3D axisymmetric model), (c) Transverse and longitudinal strain on gut surface and on gut axis for two values of the Poisson ratio ν=0.35 and ν=0.49, as a function of longitudinal position along the gut cylinder.
